# Multiomic Analysis of iPSC‐Derived Neural Spheroids Reveals Cell‐Type and Ancestry‐Specific Regulatory Landscapes of Alzheimer's Disease GWAS Genes

**DOI:** 10.1002/alz70855_104353

**Published:** 2025-12-24

**Authors:** Aura M Ramirez, Luciana Bertholim Nasciben, Sofia Moura, Lauren E. Coombs, Maria C Robayo, Farid Rajabli, Brooke A. DeRosa, Patrice G Whitehead, Larry D Adams, Takiyah D. Starks, Pedro R Mena, Maryenela Z. Illanes‐Manrique, Sergio J. Tejada, Goldie S Byrd, Mario Cornejo‐Olivas, Briseida E. Feliciano‐Astacio, Liyong Wang, Wanying Xu, Fulai Jin, Margaret Pericak‐Vance, Derek M. Dykxhoorn, Anthony J Griswold, Juan I Young, Jeffery M Vance

**Affiliations:** ^1^ University of Miami Miller School of Medicine, Miami, FL, USA; ^2^ John P. Hussman Institute for Human Genomics, University of Miami Miller School of Medicine, Miami, FL, USA; ^3^ Maya Angelou Center for Health Equity, Wake Forest University School of Medicine, Winston‐Salem, NC, USA; ^4^ Neurogenetics Research Center, Instituto Nacional de Ciencias Neurológicas, Lima, Peru; ^5^ Wake Forest University School of Medicine, Winston‐Salem, NC, USA; ^6^ Universidad Central del Caribe, Bayamón, PR, USA; ^7^ Case Western Reserve University, Cleveland, OH, USA; ^8^ Case Western Reserve University School of Medicine, Cleveland, OH, USA

## Abstract

**Background:**

Genome‐wide association studies (GWAS) have identified numerous genetic variants associated with Alzheimer's disease (AD), yet their effect sizes vary across populations. This variability stems from ancestry‐dependent differences in the genomic regulatory architecture (GRA), which governs gene expression in a cell‐type‐specific manner. Notably, the influence of local ancestry on AD risk is particularly pronounced in *APOE4* carriers. Here, we investigate ancestry‐ and cell‐type‐specific regulatory landscapes of AD GWAS genes using iPSC‐derived neural spheroids from individuals of African, Amerindian, or European ancestry.

**Method:**

Peripheral blood mononuclear cells (PBMCs) from AD patients and cognitively healthy controls were selected based on >85% global ancestry from a specific background. The PBMCs were reprogrammed into a total of 18 induced pluripotent stem cell (iPSC) lines, 6 from each ancestry, and differentiated into three‐dimensional neural spheroids containing astrocytes, neurons, oligodendrocyte precursor cells (OPCs), and oligodendrocytes. On day 76, nuclei were isolated for multiomic profiling, including single‐cell ATAC‐seq, single‐cell RNA‐seq, and bulk Hi‐C. Data were analyzed to identify ancestry‐dependent GRA differences across cell types.

**Result:**

The highest number of differentially expressed AD GWAS genes (DEAGG) was observed in astrocytes, followed by neurons, OPCs, and oligodendrocytes. Comparisons involving Amerindian ancestry samples revealed the most DEAGG across all cell types. *APOE* and *PSEN2* were differentially expressed in both astrocytes and neurons. Additional AD‐associated genes, including *SORL1* and *TMEM106B*, were differentially expressed in multiple comparisons.

**Conclusion:**

Our findings underscore the critical role of ancestry in shaping the regulatory landscapes of AD‐related genes, particularly in astrocytes and neurons. By expanding our understanding of ancestry‐specific GRA, this study provides crucial insights into the genetic mechanisms underlying AD risk and offers a foundation for more and innovative precision medicine approaches.

